# Protective Mechanisms Against DNA Replication Stress in the Nervous System

**DOI:** 10.3390/genes11070730

**Published:** 2020-06-30

**Authors:** Clara Forrer Charlier, Rodrigo A. P. Martins

**Affiliations:** Programa de Biologia Celular e do Desenvolvimento, Instituto de Ciências Biomédicas, Universidade Federal do Rio de Janeiro (UFRJ), Rio de Janeiro 21941-902, Brazil; clara.forrer.charlier@gmail.com

**Keywords:** genome stability, neurologic disease, DNA damage, neurodevelopment, neurodegeneration, ATR, CNS, replication stress, DDR

## Abstract

The precise replication of DNA and the successful segregation of chromosomes are essential for the faithful transmission of genetic information during the cell cycle. Alterations in the dynamics of genome replication, also referred to as DNA replication stress, may lead to DNA damage and, consequently, mutations and chromosomal rearrangements. Extensive research has revealed that DNA replication stress drives genome instability during tumorigenesis. Over decades, genetic studies of inherited syndromes have established a connection between the mutations in genes required for proper DNA repair/DNA damage responses and neurological diseases. It is becoming clear that both the prevention and the responses to replication stress are particularly important for nervous system development and function. The accurate regulation of cell proliferation is key for the expansion of progenitor pools during central nervous system (CNS) development, adult neurogenesis, and regeneration. Moreover, DNA replication stress in glial cells regulates CNS tumorigenesis and plays a role in neurodegenerative diseases such as ataxia telangiectasia (A-T). Here, we review how replication stress generation and replication stress response (RSR) contribute to the CNS development, homeostasis, and disease. Both cell-autonomous mechanisms, as well as the evidence of RSR-mediated alterations of the cellular microenvironment in the nervous system, were discussed.

## 1. Relevance of Genomic Stability for the Nervous System

The maintenance of genomic stability is crucial for human health. In proliferating cells, precise DNA replication and the successful segregation of chromosomes are essential for the accurate transmission of genetic information to daughter cells. Not only cell-exogenous genotoxic agents can be deleterious to genomic maintenance, but the duplication of the genome itself can create—in the wrong circumstances—a burden that can impact the genomic integrity [1,2]. Alterations in the dynamics of genome replication, also referred to as DNA replication stress, may lead to DNA damage and, consequently, mutations and/or chromosomal rearrangements. Extensive research work has established that replication stress is a source of genomic instability that may compromise the transmission of genetic information [3,4]. Appropriate replication stress response (RSR) is relevant in various biological contexts of cell proliferation—in particular, during development and in cancer [5,6,7]. In non-replicating cells, a vast machinery of genome maintenance is required to prevent and repair DNA lesions that frequently occur. Defective DNA repair pathways lead to genomic instability in postmitotic cells and are associated with aging and various neurological diseases [8,9,10].

Genetic studies of inherited syndromes have established a clear connection between mutations in DNA damage response (DDR) genes and several human diseases [11]. The immune and nervous systems are particularly susceptible to defective DDR, and the central nervous system (CNS) is severely affected when responses to threats to the genome are inadequate. Appropriate DDR is critical for developmental processes, physiological homeostasis, and for the prevention of maladies related to aging, including cancer and neurodegenerative diseases [12]. Ataxia-telangiectasia (A-T) is the most classical example of a genome instability disorder that links defective DDR and CNS diseases. Mutations in ataxia-telangiectasia-mutated (ATM) cause a severe syndrome characterized by a hypersensitivity to ionizing radiation, neurodegeneration, and ataxia. Highlighting the importance of genomic integrity, several other inherited syndromes that affect the CNS development and function are caused by mutations in genes involved in the generation or in the responses to replication stress [3]. In addition, studies in animal models have confirmed the relevance of the mechanisms that protect the nervous system against DNA replication stress [12].

Replication stress may generate genomic instability in the developing nervous system, and the importance of the replication stress response (RSR) pathways for the CNS formation has been demonstrated by many studies [13,14,15,16,17,18]. In addition, evidence that the RSR alters the cellular microenvironment and regulates immune responses has also accumulated rapidly [19,20]. Interestingly, recent research indicates that this non-cell-autonomous arm of the RSR may also contribute to the progression of neurodegenerative diseases [21].

While other reviews have broadly discussed genomic stability in the nervous system [10,12,22], here, we will focus on studies that have contributed to our current understanding about how replication stress is generated across the diverse cell types in the CNS, what players mediate RSR, and what the consequences are for CNS development and homeostasis when the RSR is defective.

## 2. Overview of DNA Replication Stress: How Cells Prevent and Respond

DNA replication occurs during the S-phase of the cell cycle. Importantly, before the actual synthesis of nascent DNA strands, key events must occur: origin licensing and replisomes formation [23]. Origin licensing takes place during late mitosis (M-phase) and the early G1 phase. Then, during the transition between the G1 and S phases, licensed origins are activated, and replisomes are formed. During the S-phase, origin firing occurs, and DNA polymerases initiate the incorporation of deoxyribonucleoside triphosphates (dNTPs) complementary to the parental DNA molecule as replication forks progress bidirectionally in opposite directions in thousands of chromosomal sites. Importantly, in normal conditions, a major fraction of licensed replication origins is not activated and remains dormant. In special conditions, such as DNA replication stress, these dormant origins may be activated [24,25,26]. 

DNA replication stress, also known as replicative stress, is defined as the slowing or stalling of replication fork progression during the synthesis of DNA [27]. Continued replication stress may lead to replication fork collapse and DNA damage that may cause mutations, amplifications, deletions, and/or chromosomal rearrangements. DNA replication stress may be caused by both cell-endogenous or cell-exogenous sources [2,3,4,7,28], and, importantly, DNA replication stress is now widely recognized as a cancer hallmark [6].

**Figure 1:**
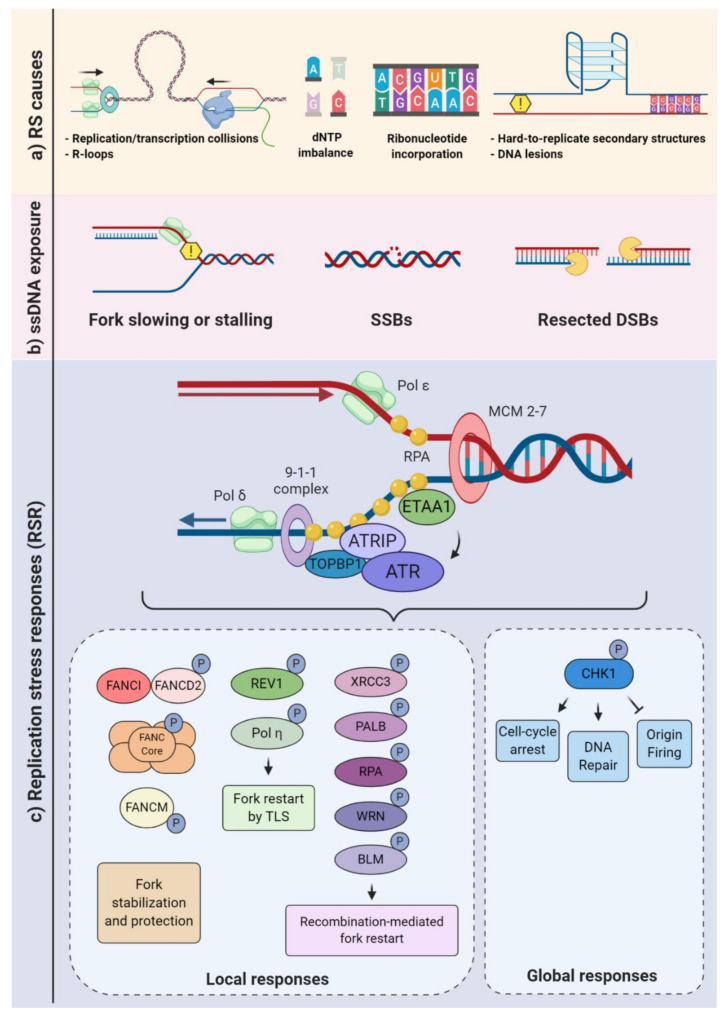
Replication stress causes and responses. (**a**) Different endogenous sources of replication stress may either slow or block the progression of the replication forks. Transcription-replication collisions (TRCs) and hard to replicate DNA are examples of obstacle challenges, and a decreased pool of nucleotides (dNTPs) is a type of metabolic imbalance. Repair of misincorporated ribonucleotides may also impair fork progression. (**b**) Replisome slowing or stalling may lead to single-stranded (ss)DNA exposure, single-strand breaks (SSB), and fork collapse. Such constraints, as well as double-strand break (DSB) resections, can result in ssDNA overhangs. (**c**) Replication protein A (RPA) binds to ssDNA, triggering the recruitment of the ATR-interacting protein (ATRIP)-ataxia-telangiectasia and Rad3-related kinase (ATR) complex, either through an interaction with DNA topoisomerase 2-binding protein 1 (TOPBP1) or Ewing’s tumor-associated antigen 1 (ETAA1). Once activated, ATR can phosphorylate various substrates generating local “at the forks” responses (e.g., fork stabilization, protection, and restart) and global responses that broadly affect the cell physiology, aimed at replication stress mitigation and/or resolution (lower panels).

Obstacles to replication fork progression, limiting or unbalanced metabolic conditions, conflicts between DNA replication and transcription machineries, and inappropriate origin firing due to oncogene activation are among the best-characterized causes of replication stress [4]. Known obstacles to replication fork progression include intrinsic characteristics of DNA sequences such as microsatellites, minisatellites, and long terminal repeats. Complex structural arrangements of the DNA molecule may also constitute a challenge (e.g., intramolecular triplex DNA, hairpins, and G-quadruplexes) to replication fork movement. DNA lesions, such as interstrand crosslinks (ICL), apurinic/apyrimidinic (AP) sites, and bulky adducts, may also slow or stall replication [2,29] (Figure 1a). 

Limiting or unbalanced metabolic conditions can also be a contributing factor in replication stress. For example, an unbalanced availability of dNTP can greatly affect the fork progression [30]. During the S-phase, the cell needs to tightly control the dNTP usage. Defective scavenging and/or de novo production may deplete the dNTP pools and very rapidly impact the fork progression [31,32] (Figure 1a). Ribonucleotide reductase (RNR) converts ribonucleotide diphosphate (NDP) to deoxyribonucleotide diphosphate (dNDP), and the NDP kinase (NDPK) converts it to dNTP [33] Conversely, the rate of degradation of the dNTPs may also be impacted by DNA replication dynamics. For example, the dNTP hydrolase SAMHD1 (Sterile alpha motif [SAM] and histidine-aspartic [HD] domain containing deoxynucleoside triphosphate triphosphohydrolase 1) is a major regulator of dNTP pools and, also, plays an important role in the RSR, with relevant implications to human health. SAMHD1 mutations were associated with cancer development and Aicardi-Goutières syndrome, a severe congenital inflammatory disease [34,35,36]. In addition, deoxynucleoside kinases and 5′-deoxynucleotidases contribute to regulate dNTP pools [33,37].

When transcription and replication machineries encounter, transcription-replication collisions (TRCs) occur. TRCs and the consequent topological stress can also be hindrances that physically impair replisome progression. At the interface of transcription and replication, one structure of particular relevance to replication stress generation is the R-loops. These nucleic acid structures are formed when a RNA strand invades the double-strand (ds)DNA, forming a DNA:RNA hybrid and a nonpaired single-stranded DNA (ssDNA) [38,39,40,41] (Figure 1a). In addition, R-loops may also occur when a RNA strand invades a single-strand break (SSB) or a double-strand break (DSB), potentially impairing the repair process [42,43,44]. Physiologically, such structures are thought to regulate gene transcription, its termination, and gene silencing [45,46,47]. However, when the R-loop formation is deregulated, it may stall or even collapse the replication fork [48,49,50]. Interestingly, TRCs can be exacerbated by R-loops [51,52,53]. Increased fork pausing and higher genomic instability were associated with head-on TRCs (when the machineries are moving towards one another) [54,55,56]. TRCs are especially relevant for genome regions that are highly transcribed during the S-phase [52,57,58,59]. Importantly, it has been suggested that TRCs are a relevant contributing factor for the occurrence of recurrent DNA double-strand breaks (DSBs) following replicative stress in neural progenitor cells (NPC) [17].

Finally, seminal studies demonstrated that inappropriate origin firing due to oncogene activation directly disturbs DNA replication, being a relevant source of replication stress [60,61,62,63]. Several mechanisms for oncogene-induced replication stress have been described. For example, oncogene activation may directly interfere with nucleotide biosynthesis, depleting dNTP pools and ceasing fork progression [64,65]. Defective origin firing and the induction of DNA re-replication are also among the outcomes of replication stress caused by oncogenic stimulus [66,67]. 

The replication stress response is broadly defined as a branch of the DNA damage response that specifically reacts to DNA replication stress, embracing multiple signaling pathways and the downstream cellular responses. As previously characterized for other subdivisions of DDR, the cellular responses to replication stress are diverse and may be subdivided into local and global responses. Local responses are the mechanisms that take place at the replication forks, whereas global responses include pan-nuclear and cytoplasmic processes, as well as functional responses that regulate the cellular microenvironment [19,68]. The stabilization of stalled forks, promotion of fork restarts, and regulation of origin firing are examples of processes that directly regulate replication forks or dormant origins. The activation of DNA repair mechanisms and checkpoints that inhibit cell cycle progression, as well as cell death, senescence, and cytokine production pathways, are among the well-characterized global responses to replication stress [3,4] (Figure 1). 

Upon fork slowing or stalling, one of the first molecular consequences is the exposure of ssDNA. A junction of ssDNA and double-strand DNA (dsDNA) creates a platform for the recruitment of various proteins. ssDNA tracts are coated by replication protein A (RPA) and other proteins (e.g., TOPBP1, ETAA1, and 53BP1), leading to the activation of the ATR-ATRIP complex (composed of the ataxia-telangiectasia and Rad3-related kinase and the ATR-interacting protein) (Figure 1c). Then, the ATR kinase may phosphorylate more than 700 target proteins, activating multiple signaling networks. Consequently, complex responses that range from cell cycle arrest, the regulation of origin firing, replication fork stabilization, replication fork restart, and the control of dNTP availability are elicited. Notably, ATR-mediated phosphorylation and the activation of checkpoint kinase 1 (CHK1) are extremely relevant to regulate both local and global responses [69,70,71,72].

**Figure 2:**
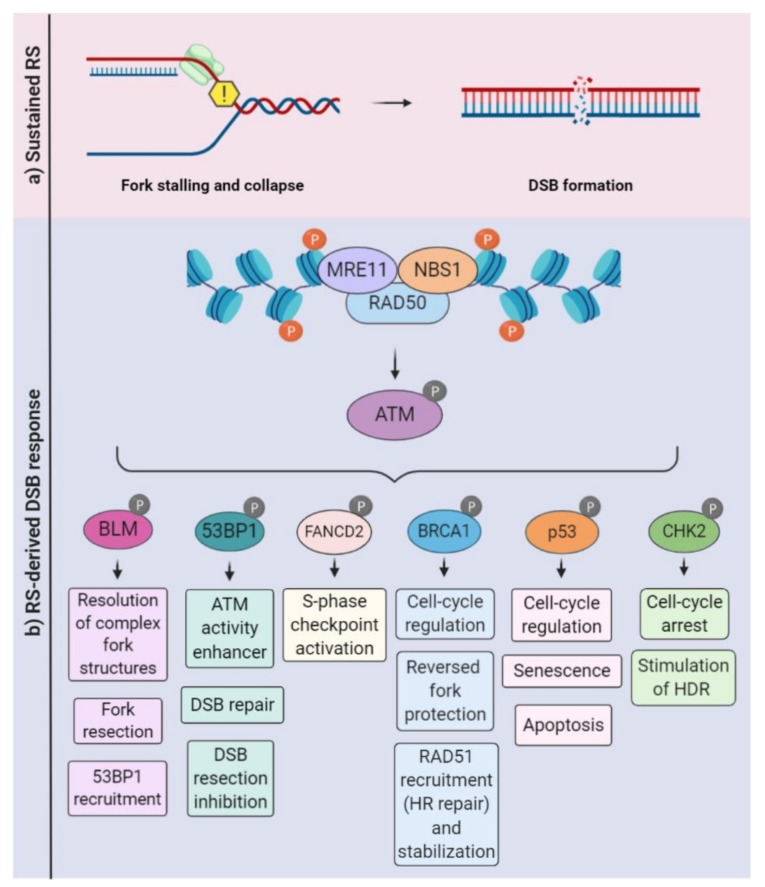
Ataxia-telangiectasia-mutated (ATM)-mediated signaling in the context of replication stress. (**a**) Sustained replication stress can induce fork collapse and DSBs, leading to the recruitment of the MRN (MRE11, RAD50, and NBS1) complex. (**b**) The MRN complex associates with the DSB, recruiting and activating ATM. Depending on specific scenarios (e.g., intensity and duration), ATM phosphorylates a variety of substrates and coordinates a variety of cellular responses, including DSB repair, the inhibition or stimulation of DNA resection, the activation of cell-cycle checkpoints, and cell death programs. HDR: homology-directed repair.

Replication fork stabilization and fork restart, as well as fork reversal, contribute to prevent the replication fork collapse, which is a known source of DNA double-strand breaks (DSBs), an extremely cytotoxic lesion. The generation of DSBs triggers the activation of other signaling kinases, including ataxia-telangiectasia-mutated (ATM) and DNA-PKcs (DNA-dependent protein kinase, catalytic subunit). It is well-established that there is a considerable overlap in the activation of these kinases following continued replication stress and the consequent DSBs (Figure 2). For example, depending on the DNA repair pathway elicited, the resection of DSBs exposes ssDNA that fall under the purview of ATR [11,73]. Classical and alternative DNA repair pathways, such as homologous recombination, nonhomologous end joining (NHEJ), or break-induced replication repair, may be employed to allow fork restart [74,75,76]. When repair is not possible, the mechanisms of damage tolerance may be activated to restart the fork, such as the lesion bypass (where the replisome “skips” the lesion and resumes replication downstream), template switch (a homologous recombination (HR)-mediated response that involves fork regression and the use of the newly synthesized DNA strand as a template) and translesion synthesis (TLS), in which specialized polymerases capable of bypassing the lesion are recruited [77,78,79].

There is a plethora of factors involved in the attenuation or repair and rescue of replication stress. In addition to these autonomous cellular responses, it is now well-documented that replication stress may also induce dramatic alterations in the cellular microenvironment. This non-cell-autonomous paracrine arm of RSR occurs through the regulation of proinflammatory cytokines [19,80]. In fact, a series of elegant studies provided evidence for replication stress-induced inflammation as a driver of neuronal degeneration in models of ataxia telangiectasia. Studies about the immune response in *At*^−/−^-mice revealed that the accumulation of cytoplasmic DNA in microglial cells triggered the release of neurotoxic cytokines, leading to chronic neuroinflammation and neurodegeneration [81,82]. Understanding the contributions of RSR to nervous system development, degeneration, and regeneration is of fundamental biomedical importance. In the next section, we provide an overview of biological contexts when cell proliferation and replication stress may occur in the nervous system physiology and pathology, focusing on humans and mouse models.

## 3. Cell Proliferation in Nervous System Development, Homeostasis, and Diseases

The process of DNA replication is essential for cell proliferation, and the dynamics of cellular renovation vary significantly in multicellular organisms [83]. While some tissues exhibit a high rate of cell proliferation throughout life, in various CNS tissues, progenitors undergo a burst of cell proliferation during development and have a very limited generation of new cells afterwards [84,85]. It is estimated that, during development, the human brain gives rise to 160 billion neuronal and glial cells [83,84,85,86].

**Figure 3:**
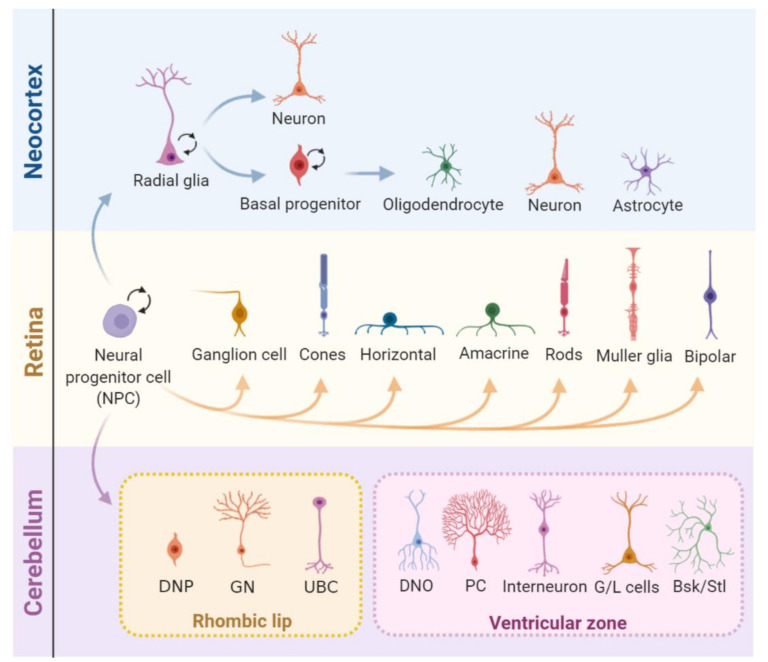
Cell proliferation during the central nervous system (CNS) development. In the developing neocortex, first, the neural progenitor cells (NPCs) undergo expansion and, later, shift identity to radial glia (RG) cells). These can divide either symmetrically, expanding its pool, or asymmetrically, generating either a combination of a radial glial cell and a neuron or a radial glial cell and a basal progenitor (BP). BPs can further divide symmetrically into two BPs or asymmetrically into neurons or glia (oligodendrocytes and astrocytes). After embryonic day 15.5 (E15.5), the radial glia progressively loses its neurogenic potential in favor of gliogenesis. The mature retina is composed of seven major cell types that derive from multipotent progenitor cells. These undergo unidirectional shifts in their competence in tightly controlled timeframes, generating multiple neuron types and Müller glia. In the cerebellum, two pools of NPCs that originate from different regions of the embryo give rise to different types of neurons and glia. Cerebellar NPCs that originate in the upper rhombic lip (RL) form the external granule layer and give rise to glutamatergic neurons, and NPCs from the ventricular zone (VZ) originate GABAergic neurons. DNP: deep nuclear neurons, GN: granule neurons, UBC: unipolar brush cells, DNO: deep nuclei olivary neurons, PC: Purkinje cells, G/L cells: Golgi and Lugaro cells, and Bsk/Stl: basket/stellate cells.

Developmental neurogenesis relies on the rapid expansion of neural progenitor cell (NPC) pools [87]. Murine cortex neurogenesis occurs during the embryonic stages and is already finished at birth. First, neural progenitor cells (NPCs) undergo symmetric divisions (generate two stem cells) that expand the progenitor pool until embryonic day 10 (E10). Upon the start of neurogenesis, asymmetric divisions of NPCs with astroglial features (also known as radial glia) initiate in the ventricular zone (VZ) [88]. Depending on the environmental cues, these NPCs can either generate a postmitotic neuron and a NPC or intermediate progenitors that locate to the subventricular zone (SVZ) and can further divide into neurons, astrocytes, or oligodendrocytes [89]. Later, a switch from neurogenesis to gliogenesis generates astrocytes and, after birth, oligodendrocytes [90,91,92,93,94]. The retina is another well-studied CNS tissue that is also affected in human diseases caused by mutations in DDR genes [95,96]. In mice, retinogenesis starts during embryonic development and extends until postnatal ages. Importantly, all neuronal cell types and the Muller glial derive from a pool of multipotent retinal progenitor cells [97,98], but retinal astrocytes derive from a distinct progenitor pool that migrates into the retina through the optic nerve after birth [84,99]. Finally, different from the neocortex and the retina, the cerebellum originates from two distinct populations of NPCs that occupy different germinative areas: the ventricular zone (VZ) and the rhombic lip (RL). In mice, its development also expands from embryonic stages to later postnatal developments [84,100,101] (Figure 3).

**Figure 4:**
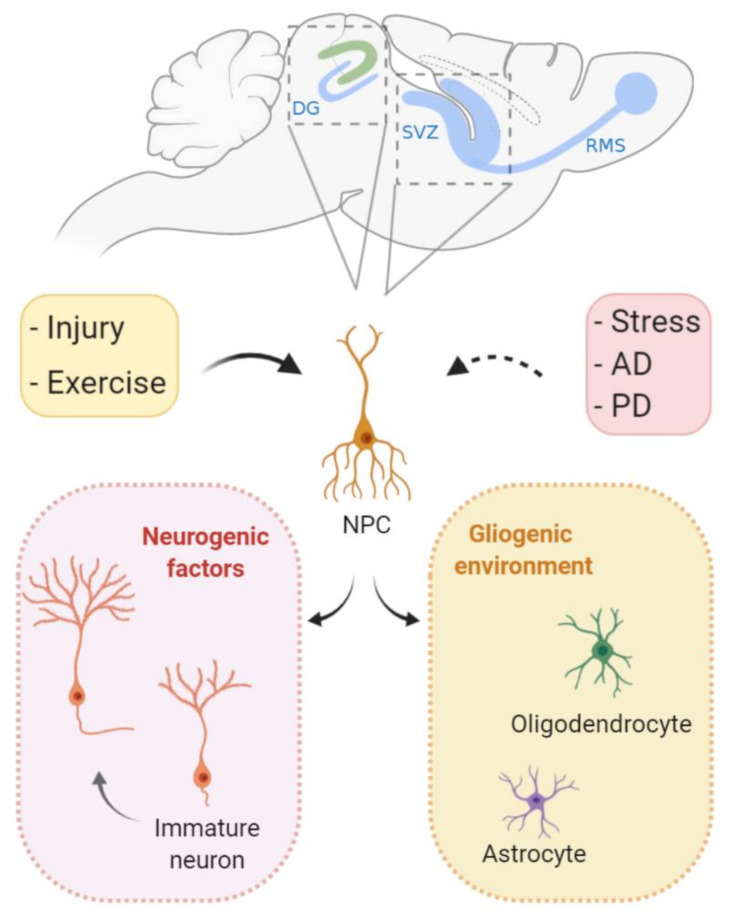
Cell proliferation during adult neurogenesis. In specific brain regions, neural stem cells give rise to functional neurons in the mature brain. The subventricular zone (SVZ) and the dentate gyrus (DG) of the hippocampus are the two main sites of adult neurogenesis. Following environmental cues, gliogenic programs can lead to terminal differentiation in glial cells (oligodendrocytes and astrocytes). In specific occasions, neurogenic factors direct proliferating stem cells to differentiate into neurons that migrate and integrate with previously established neuronal circuits. It is believed that, during Alzheimer’s and Parkinson’s disease, cell proliferation and neurogenesis are inhibited in the mature brain. In contrast, physical activity can induce neurogenesis. Interestingly, even though injury itself leads to cell loss, it may also induce cell proliferation both locally and nonlocally. RMS: rostral migratory stream, AD: Alzheimer’s disease, and PD: Parkinson’s disease.

Even though cell proliferation is largely limited to development in mammal CNS, it can also occur during adulthood. The two main sites of adult neurogenesis are the subventricular zone (SVZ) and the dentate gyrus of the hippocampus [102,103]. The functions of these adult-born neurons are still a highly contentious subject, and potential functions include memory acquisition and/or loss [104,105,106]. Nevertheless, despite the putative functions, the presence of proliferation into adulthood exposes proliferating cells to the risk of replication stress and its possible deleterious consequences (Figure 4).

Replication stress is a driver of genomic alterations that are required for cellular transformation [6], and, regardless of the cellular origin, unscheduled replication is a hallmark of tumorigenesis [107]. Glioblastoma, one of the most lethal human cancers, medulloblastoma, the most common pediatric brain tumor, and the pediatric cancer retinoblastoma are among the most studied CNS tumors [108,109,110,111]. The progressions of these CNS tumors depends on unrestricted cell proliferation, and replication stress response pathways are often deregulated in these diseases [112,113,114]. Absent or defective checkpoints contribute to enhanced cell proliferation, the prevention of apoptosis, and other relevant transformation processes. For example, it was proposed that replication stress is responsible for the upregulation of DDR factors and, consequently, for the radiation resistance of glioma stem cells [115,116,117]. Recent therapies that combine DNA-damaging agents and target replication stress mediators have been intensively studied as a therapeutic strategy, including in CNS tumors [118,119,120].

## 4. Replication Stress-Causing Factors in the Nervous System

### 4.1. DNA Polymerases

The fidelity of DNA replication depends on the selection of correct and undamaged nucleotides for incorporation by DNA polymerases. DNA polymerases exhibit differences in processivity and fidelity, and at least nine of them are involved in the replication of the nuclear genome. Polymerases α, delta e, and epsilon perform the vast majority of nuclear DNA replication and nearly always insert correct dNTPs [25]. When replicating forks encounter DNA lesions that distort the double helix, the above-mentioned polymerases involved in regular DNA replication are blocked. In this context, DNA-damage bypass pathways, such as translesion synthesis, template switching, homologous recombination, and repriming, can be employed [77]. Polymerases zeta, eta, iota kappa, and Rev1 are among the best-characterized translesion synthesis (TLS) polymerases, but others such as pol β also exhibit translesion activity. In addition to its TLS activity, DNA polymerase β is a key enzyme for the base excision repair (BER) pathway [121].

*Polβ* gene-null mice die perinatally and exhibit severe nervous system defects mainly due to apoptosis in the CNS and PNS, but the cell deaths of replicating NPCs were, at first, not evaluated [122]. Later, the conditional inactivation of Polβ using distinct forebrain-specific Cre lines revealed frequent DSBs in cortical NPCs during the S-phase, likely due to defective BER in these progenitors [123]. The possible contributions of Polβ-mediated translesion activities and replication stress to the death of these progenitor cells were not considered. While the roles of translesion DNA polymerases in replication stress have been studied in multiple biological contexts [124], including DNA repair [125], their contributions to protective mechanisms against DNA replication stress in the nervous system remain unexplored. 

### 4.2. RecQ Family of DNA Helicases

DNA helicases of the RecQ family have been shown to play a role in replication stress [126], particularly in the resolution of replication intermediates and arrested forks. Mutations in genes of this family cause three related syndromes: Bloom (BLM), Werner (WRN), and Rothmund-Thomson (RECQL4), which lead to phenotypes in the nervous system.

#### 4.2.1. WRN

Werner syndrome (OMIM 277700) is a rare autosomal recessive progeroid disorder in which patients exhibit accelerated aging, bilateral cataracts, diabetes mellitus, osteoporosis, and a predisposition to rare cancers [127,128]. Although a neurological disease is not a classical feature of Werner’s syndrome patients, brain atrophy (~40% of the patients), altered memory, and neuropathies were reported [129,130] (Table 1).

The WRN gene (or RECQL2) encodes a RecQ DNA helicase that possesses both exonuclease and 3′ to 5′ helicase activities and has reported roles in replication fork functions, either in the prevention or resolution of the fork collapse, as suggested by DNA fiber studies [128]. The prevention of excessive resection in replication stress-induced stalled forks was also reported [131,132,133]. In addition, WRN interacts with class I histone deacetylase (HDAC1), protecting cells from hydroxyurea-induced fork arrest [134] (Figure 1).

At the cellular level, mitochondrial dysfunction with excessive ROS production [135], increased gene methylation [136,137], premature telomere shortening [138], and the decreased proliferation of stem cells were observed [136]. An analysis of WRN mice models suggested that microglial dysfunction, altered levels of inflammatory cytokines, and neuronal oxidative stress may account for some of the neurological symptoms [135,136,137,138,139].

#### 4.2.2. BLM

Bloom syndrome (OMIM 210900) is an autosomal recessive disorder, also named microcephaly, growth restriction, and increased sister chromatid exchange-1 (MGRISCE1) [140]. Bloom syndrome patients’ features include growth defects, microcephaly, decreased intellectual ability, immunodeficiency, retinopathies, skin abnormalities, infertility, and a predisposition to hematological malignancies. The disease is caused by homozygous or compound heterozygous mutations in *BLM*, the gene encoding DNA helicase RecQ protein-like-3 (RECQL3) [140,141]. 

BLM presents multiple functions in genome maintenance and replication. Depending on the phase of HR, it can have pro- or anti-recombinogenic activity (e.g., stimulating a RAD51 homology search and strand invasion or the dissolution of the D-loop in later stages) [142,143,144] (Figure 1 and Figure 2). It has a unique function amongst the RECQ helicases: the ability to resolve ultra-fine bridges (UFBs) following chromatid segregation [145]. The maintenance of fork stability during replication, dealing with structures such as G quadruplexes, was also described [146]. Another proposed function of BLM in the prevention of replication stress came from the observation that BLM-deficient cells have an increase in fork stalling and are hypersensitive to replication stress induction [147,148]. In addition, during replication stress, BLM is targeted to non-centromeric abnormal structures and cooperates with the FANC pathway proteins to prevent and resolve sister chromatid bridging, avoiding micronuclei and aneuploidy [149]. In mice, the inactivation of *Blm* is early-embryonic lethal [150], and its heterozygosity or hypomorphism predisposes to tumorigenesis. No CNS phenotype was described, and no CNS-specific inactivation has yet been published.

#### 4.2.3. RECQL4

Rothmund-Thomson syndrome (OMIM 268400) is a very rare recessive autosomal disease [151]. Clinical manifestations of the syndrome include skin depigmentation, hypogonadism, alopecia, short stature, juvenile cataracts, microphthalmia, microcornea, glaucoma, cognitive deficits, and, eventually, cerebral atrophy [152,153]. A high predisposition to neoplasias—specially, osteosarcoma—was also reported [154] (Table 1).

The RECQL4 protein participates in HR and NHEJ DSB repair [155,156] and telomere maintenance [157]. Its roles in origin activation during the S-phase and chromosome alignment during replication were also described [158,159,160,161]. Different mice models of *Recql4* inactivation have been generated, but no CNS phenotypes were found [162,163,164].

### 4.3. Senataxin, Spinocerebellar Ataxia with Axonal Neuropathy 2, and Amyotrophic Lateral Sclerosis 4

Mutations in the SETX gene that encodes the protein senataxin are associated with two rare autosomal diseases of distinct inheritance: spinocerebellar ataxia, autosomal-recessive, with axonal neuropathy 2 (SCAN2, formerly known as AOA2, OMIM #606002), and amyotrophic lateral sclerosis 4 (ALS4, OMIM #602433), a juvenile form of ALS (Table 1). Despite sharing the same gene as a cause, SCAN2 is an autosomal-recessive disease characterized by loss-of-function mutations, while ALS4 is an autosomal-dominant trait associated with gain-of-function alterations [47,165,166,167,168]. SCAN2 clinical manifestations include progressive cerebellar atrophy, ataxia, and sensorimotor peripheral neuropathy [169,170,171,172,173]. In contrast, ALS4 mainly affects the motor neurons and the spinal cord, leading to muscle dysfunction [174,175,176].

Senataxin is an RNA/DNA helicase, with several roles in transcription dynamics. Senataxin-deficient cells present a decreased association of RNA polymerase II with several gene loci and undergo premature termination [177,178,179]. An increased sensitivity to genotoxic agents and increased DSB formation were also reported, indicating defective DNA repair following senataxin LOF [180,181]. In yeast, senataxin associates with replication forks and promotes their progression across RNA polymerase II-transcribed genes, coordinating the transcription and replication [182]. Consistently, it was observed that the formations of senataxin and 53BP1 foci were proportional to the degree of replication stress induced [183]. Recently, it was shown that senataxin is recruited to DSB formed in transcriptionally active genes. Even though it did not seem to be involved in the resolution of R-loops, the promotion of the Rad51 foci formation and the inhibition of translocation following DSB induction were reported [184].

*Setx* knockout mice failed to replicate the neurological phenotypes found in SCAN2 patients but revealed interesting insights into senataxin functions. In vivo LOF led to infertility, the failure in meiotic sex chromosome inactivation (MSCI), R-loop accumulations, DSBs, and the defective dissociation of Rad51 filaments [185]. Later, it was suggested that spermatogenesis defects were caused by reduced SUMOylation and the impaired recruitment of ATR and CHD4 to the XY body following senataxin loss [186]. Notably, the R-loop accumulations were not observed in the brain. Other mice models aimed to recapitulate the Setx gain-of-function as found in ALS4, showing the progressive degeneration of motor neurons and other neuromuscular phenotypes [176]. We did not find studies about the CNS-specific inactivation of senataxin or brain organoids models of these diseases.

### 4.4. Aicardi-Goutières’ Syndrome-Causing Genes

Aicardi-Goutières syndrome (AGS) is a genetically heterogeneous encephalopathy. AGS patients’ clinical manifestations include cerebral atrophy, intracranial calcification, and leukodystrophy, as well as increased interferon alpha (α-IFN, *IFNA1*) and leukocytosis in the cerebrospinal fluid [187]. Progressive microcephaly, psychomotor retardation associated with the demyelination of motor neurons, and death in early childhood are also common. These neurological manifestations are associated with mutations in seven different genes (*TREX1, SAMHD1, RNASEH2A, RNASEH2B, RNASEH2C, ADAR1,* and *IFIH1*) [187,188,189,190] (Table 1). The severity and onset of the disease correlate with the gene mutated, the *TREX1* usually being the most severe manifestation [191,192].

#### 4.4.1. TREX1 Exonuclease

TREX1 (Three prime Repair Exonuclease 1), previously designated DNase III, is a dsDNA and ssDNA 3′-5′ exonuclease that has important roles in DNA repair and the degradation of foreign DNA that reaches the cytoplasm [193]. TREX1 loss-of function (LOF) leads to accumulation of self-DNA and RNA, which can trigger a potent immune reaction [189,194,195]. *Trex1*-null mice show a reduced lifespan due to the development of serious inflammatory cardiomyopathy but are viable [196]. Subsequent work has revealed that much of the chronic immune response triggered by cytoplasmic DNA in Trex1 knockout (KO) was due to the reverse transcription of the retrotransposon LINE-1 (Long INterspersed Element 1). As shown in *TREX1*-deficient cell lines, *Trex1*-null mice, and patient-derived organoids, TREX1 inhibits LINE-1 translocation across the genome [197,198,199]. In fact, such suppressions of LINE-1 retrotransposition prevent an increased interferon I secretion, a hallmark of AGS [199]. Morita and colleagues reported no neurological symptoms in *Trex1*-null mice, but later inflammatory signatures were reported in many different organs, including the brain [196,198,200]. Cell-type-specific inactivations of *Trex1* in either NPCs or the microglia did not cause the mild brain inflammation of the full KO, but the microglia-specific inactivation of *Trex1* caused a spontaneous interferon response in the CNS [201]. 

#### 4.4.2. SAMHD1

SAMHD1 (Sterile alpha motif [SAM] and histidine-aspartic [HD] domain containing deoxynucleoside triphosphate triphosphohydrolase 1) is a dNTP hydrolase that depletes dNTP pools in the cytoplasm [202]. SAMHD1 also exhibits 3′ exonuclease activity against RNA and DNA, promotes end resection, and facilitates DSB repair by HR [203,204,205]. Primary SAMHD1-deficient fibroblasts from AGS patients present chronic DDR activation and elevated type I IFN levels [206]. Recently, it was shown that SAMHD1 acts directly in stalled forks, stimulating the exonuclease activity of MRE11 and limiting the accumulation of cytoplasmic ssDNA, which may induce proinflammatory type I interferons [36]. In fact, *Samhd1*-null mice do present the constitutive IFN production, but no evidence of brain inflammation, as observed for *Trex1* knockout, or neurological phenotypes were reported [207].

#### 4.4.3. RNAse H Ribonucleases and RNA Deaminase (ADAR1)

Mutations in RNase H2 (RNA:DNA hybrid-specific ribonuclease H2 subunit) are the most common causes of AGS. RNASEH2 is a ribonuclease that cleaves the 5′-phosphodiester bond of ribonucleotides embedded in a dsDNA (RNA:DNA hybrids), mediating the excision of a single ribonucleotide embedded in genomic DNA (gDNA) and the removal of R-loops [41,208]. As observed in AGS patients harboring *TREX1* mutations, RNASEH2-mutated cells also accumulate cytoplasmic DNA. However, its origin is not clear, because, in contrast to TREX1, RNASEH2 is thought to facilitate retrotransposon mobility [209]. *Rnase2b*-null mice had either embryonic or perinatal deaths and accumulated ribonucleotides in the gDNA, activating the DNA damage response, but did not recapitulate the nervous system impairments of AGS patients [210,211,212]. In contrast to the severe phenotype associated with the human disease, mice models coding hypomorphic RNase H2 [213,214] or a brain-specific inactivation of *Rnase2b* did not display neuroinflammation or other clinical signs [215].

ADAR1 (Adenosine Deaminase Acting on RNA) deaminates specific adenosines to inosines in dsRNA. It is well-established that ADAR1 modifies the host RNA and modulates the sensing of self- versus nonself RNA, allowing pathogen detection and preventing an autoimmune response [216,217]. However, edition-independent functions of ADAR proteins also have been demonstrated [218,219]. Disease-related ADAR1 mutations have been associated with a type 1 interferon gene expression signature [220]. Although *Adar1*-null mice are embryonically lethal and the RNA-editing activity of ADAR1 is crucial for the aberrant innate immune response, no neurological symptoms were observed [221,222]. Recent studies have shed light on the potential mechanisms of the nervous system dysfunction following ADAR1 deficiency. In cultured human cells, the differentiation of *Adar1*-deficient neuronal progenitor cells (NPCs) induced a spontaneous upregulation of IFN and IFN-stimulated genes that were mediated by the dsRNA sensor MDA5 [223]. Finally, flies expressing an editing-incapable point mutant of ADAR display locomotor deficits and neurodegeneration, indicating that the ADAR function in the CNS is editing-dependent [224].

### 4.5. CTC1 and Telomere Maintenance

Cerebroretinal microangiopathy with calcifications and cysts (CRMCC), also known as Coats Plus syndrome (OMIM 612199), is a rare autosomal multisystem disease characterized by intracranial calcifications and brain cysts, leukoencephalopathy, retinal vascular abnormalities, and other non-neurological manifestations [225,226]. Mutations in *CTC1* (CST telomere replication complex component 1) cause this syndrome [227,228]. CTC1 is one of the members of the CST complex, also composed of STN1 and TEN1, that regulates telomere replication and maintenance by facilitating the restart of stalled forks at telomeres [229]. In mice, *Ctc1* inactivation led to the loss of leading C-strand telomeres, the accumulation of single-strand telomeric DNA, and sustained ATR-mediated G2/M arrest due to an impaired fork restart [230]. A CTC1-RAD51 functional interaction was proposed as the mechanism for fork restart that would facilitate replication under stressed and unstressed conditions [231,232]. Further research is needed to determine whether CTC1 and the CST complex play a role in proliferating cells of the nervous system.

### 4.6. Fanconi Anemia-Causing Genes

Fanconi anemia (FA) is a genetically heterogeneous inherited disease resulting from mutations in the regulators of genomic stability. It is characterized by congenital abnormalities, bone marrow failures, and cancer predispositions. The nervous system features include microcephaly, brain and spinal cord abnormalities, and a medulloblastoma predisposition. However, recent clinical studies show that the incidence of CNS abnormalities in FA is higher than initially thought, and among the described alterations are pituitary and corpus callosum malformations, as well as cerebellar atrophy [233,234,235,236] (Table 1).

Pathogenic variants have been identified in at least 22 genes, including FANCA, FANCB, FANCC, FANCD1/BRCA2, FANCD2, FANCE, FANCF, FANCG, FANCI, FANCJ/BRIP1, FANCL, FANCM, FANCN/PALB2, FANCO/RAD51C, FANCP/SLX4, FANCQ/ERCC4/XPF, FANCR/RAD51, FANCS/BRCA1, FANCT/UBE2T, FANCU/XRCC2, FANCV/REV7, and FANCW/RFWD3. These genes code for a network of proteins referred to as the FA/BRCA DNA repair pathway. The canonical function described for this pathway is the removal of interstrand crosslinks (ICL) that compromise DNA replication and transcription [237,238]. Its direct roles in the replication of fork stability and the prevention of replication stress are known. Nascent DNA strands are protected by FA pathway proteins, including monoubiquitinated FANCD2, RAD51, BRCA1, and BRCA2. The regulation of dormant origin firing by FANCI, the prevention of MRE11-mediated resection on stalled forks, and contributions to the resolution of RNA:DNA hybrids (R-loops) have also been reported [48,239,240,241,242] (Figure 1 and Figure 2). In addition, it appears to be involved in chromosome maintenance (the prevention of DNA under-replication and subsequent ultra-fine bridge formation, regulation of the spindle assembly checkpoint, and the protection of fragile sites). It also cooperates with BLM to successfully separate sister chromatids [243,244,245]. Mice models for FA genes contributed to the understanding of their relevance to the nervous system development [246]. While most models do not exhibit gross developmental defects [247], some display neurodevelopmental issues: Fanca^−/−^, Fancc^−/−^, and Fancd2^−/−^. Fancg^−/−^ and Fanci^−/−^ present microphthalmia, and Fanca^−/−^ and Fancg^−/−^ display microcephaly, while Fancp^−/−^ has hydrocephalus and ocular abnormalities [248,249,250,251]. The most commonly affected gene is FANCD1/BRCA2, which is mutated in up to 5% of FA cases. The germline mutation of Brca2 is lethal [252]. The CNS-specific inactivation of Brca2 profoundly affected developmental neurogenesis due to DNA damage-induced apoptosis and caused severe microcephaly and cerebellar defects. Consistent with the occurrence of brain tumors in FANCD1-mutated patients [253], mice deficient for both Brca2 and p53 developed medulloblastoma, highlighting the importance of BRCA2 to neural development and CNS tumor suppression [254]. More recently, evidence has been shown that FANCD2 protein levels are strongly associated with the glioblastoma tumor grade, and the inhibition of the FA pathway sensitizes gliomas to chemotherapeutic agents [255].

### 4.7. XRCC1 and DNA Single-Strand Break Repair

Mutations in proteins involved in DNA single-strand break (SSB) repair cause neurodegenerative diseases [256,257,258,259] (Table 1). DNA SSBs may be generated by various different mechanisms, such as lesions caused by reactive oxygen species (ROS), base excision repair (BER) intermediates, or as a consequence of topoisomerase activity during DNA replication [260]. XRCC1 is a scaffold protein that interacts and stabilizes protein complexes that are crucial for DNA single-strand break (SSB) repair. While the germline inactivation of *Xrcc1* resulted in embryonic lethality [261], its inactivation specifically in NPCs induced microcephaly, the loss of cerebellar interneurons, and progressive ataxia [262]. A recent study showed that biallelic mutations in human *XRCC1* are associated with ocular motor apraxia, axonal neuropathy, and cerebellar ataxia [263]. In addition to defective SSB repair, the cells of this patient elevated levels of protein ADP ribosylation. Interestingly, the genetic inactivation of Parp1 (poly (ADP) ribose polymerase 1) rescued ADP ribose levels and reduced the loss of cerebellar neurons and ataxia in Xrcc1-defective mice, implying that PARP1 hyperactivation was neurotoxic to cerebellar neurons [263]. Several studies demonstrated the roles of both XRCC1 and PARP1, as well as their functional cooperation in the generation of replication stress [264,265,266].

## 5. Replication Stress Response in the Nervous System

### 5.1. PI-3 Kinases: ATR, ATM, and DNA-PK

#### 5.1.1. ATR, ATRIP, and Seckel Syndrome

Seckel syndrome is an autosomal recessive disorder characterized by intrauterine growth retardation, severe dwarfism, microcephaly, and mental retardation [267] (Table 1). Other important neurological features of Seckel patients include: cortical and retinal malformations and visual impairments associated with the lack of photoreceptor functions [268,269,270]. Mutations in distinct genes have been associated with Seckel. Centriole biogenesis, DNA damage responses, and, more broadly, genome maintenance are among the described functions of the affected genes [271].

Mutations in *ATR* (ATM and rad3-related) and in *ATRIP* (ATR-interacting protein) are among the causes of Seckel syndrome. The first link between RSR and Seckel syndrome was the identification of mutations in *ATR* [272,273]. Later, LOF mutations in *ATRIP*, which also lead to ATR protein loss, were also identified [274]. The ATR/ATRIP complex plays a crucial role in cellular responses to single-strand DNA damage and replication stress and, therefore, maintaining genomic stability [71,73,275]. At the replication fork, ATR may regulate replication origin firing, stalled fork stability, and restart. Known global responses downstream of ATR are the control of cell cycle checkpoints and the control of dNTP availability (Figure 1c). In addition, the ATR/ATRIP signaling pathway is also activated in more specific scenarios of DDR, such as its activation in telomeres during translesion synthesis, DSB, or ICL repair [3,71,73,275].

The importance of ATR-mediated RSR to the CNS development was deeply studied in mice models. Not surprisingly, the germline inactivation of *Atr* led to early embryonic lethality [276,277]. The first Atr Seckel mouse model showed that ATR LOF severely impacted the nervous system development, inducing the accumulation of DNA damage and apoptosis of NPCs in the embryonic and postnatal developments of the forebrain [278]. The genetic inactivation of *Atr* specifically in NPCs of the developing brain also led to brain growth impairments and cerebellar dysgenesis. In the cerebellum, *Atr* loss resulted in proliferation arrest, while, in the ganglionic eminence, extensive DNA damage and p53-mediated apoptosis was detected [15]. Interestingly, a blockade of apoptotic pathways through p53 inactivation did not rescue the growth impairment and neuropathology of these neural tissues [14,15]. Therefore, it remains to be determined which cellular events triggered by defective RSR are the cause of CNS malformations. In addition, it is not yet clear whether the Seckel malformations described for other CNS tissues are caused by similar molecular and cellular mechanisms. Moreover, to date, no studies have addressed the consequences of ATRIP LOF in the CNS development.

#### 5.1.2. ATM

Ataxia telangiectasia (A-T, OMIM #208900) is an autosomal recessive syndrome caused by a mutation of the ataxia-telangiectasia-mutated (*ATM*) gene [279]. It is characterized by dilated blood vessels (telangiectasias), radiosensitivity, immunodeficiency, cancer susceptibility, progressive cerebellar ataxia, and neurodegeneration [280,281] (Table 1). 

Seminal studies on DNA replication revealed that A-T patients’ cells did not stop replicating their DNA following irradiation due to defective cell cycle checkpoints [282,283]. Decades later, it became clear that ATM kinase is the master regulator of cellular responses to DSBs, including DNA repair, cell cycle checkpoints, and apoptosis, among others [73,284,285]. Some examples of ATM targets in the replication stress context can be seen in Figure 2. Importantly, in addition to its roles in DDR, alternative mechanisms of ATM activation have been described (e.g., ATM direct oxidation; see reference [286]. Moreover, ATM regulates a diverse array of cellular processes, including oxidative stress-induced responses, peroxisome maintenance, and glucose metabolism [287,288,289,290,291,292]. Therefore, the cellular and molecular mechanisms that contribute to the neurodegenerative phenotypes associated with ATM deficiency are still under intense debate (see Section 6).

To some extent, the signaling of DNA DSB and RSR have a significant overlap, and ATM and ATR kinases may be cooperatively activated. In conditions of continued stress, stalled forks may generate DSBs; therefore, replication stress may culminate in ATM activation. On the other hand, when ATM induces a homologous recombination (HR) to repair a DSB, the Mre11-mediated resection generates RPA-coated ssDNA that leads to ATR activation [293,294]. A recent study revealed that ATM and ATR cooperate to maintain genomic stability in progenitor cells of the developing brain. Consistent with a previous analysis of Atr function during unchallenged DNA replication [15], upon exposure to irradiation, it was observed that ATR mediates the G2/M checkpoint in proliferating NPCs. Since ATM was only required for DNA damage-induced apoptosis in differentiating progenitor cells, it was proposed that these kinases may have unique and nonoverlapping functions in the developing CNS [295].

#### 5.1.3. DNA-PK

Recessive biallelic mutations in the *PRKDC* gene that expresses the catalytic portion of DNA-PK (known as DNA-PKcs) cause IMD26 (Immunodeficiency 26, OMIM 615966), a syndrome that can be accompanied by neurological manifestations, including microcephaly, seizures, and hearing and vision losses. Imaging studies have revealed widespread brain atrophy, as well as hypomyelination in some areas [165,296,297] (Table 1).

The best-characterized function of DNA-PK is the repair of DSBs through the NHEJ repair pathway [73,298]. However, similar to ATR and ATM, DNA-PK interacts with proteins involved in other DDR processes. For example, DNA-PK may functionally cooperate with regulators of HR, cell cycle checkpoints, and telomeric maintenance [299,300,301,302,303,304,305,306,307,308,309]. In the context of replication stress, it was shown that ATR phosphorylates DNA-PK following UV-induced replication stress and that the DNA-PK function facilitates DNA damage resolutions [310]. The phosphorylation of DNA-PK can reinforce the ATR-Chk1-mediated DDR by promoting a claspin-Chk1 interaction stability, and it was proposed that DNA-PK may act as a signal amplifier of ATR [311,312]. Moreover, upon ATR inhibition, DNA-PK can act as a backup pathway that phosphorylates Chk1 and other targets, suppressing origin firing [299]. In addition, DNA-PK was also shown to, together with PARP1, recruit XRCC1, allowing the repair and restart of stalled replication forks [264].

Severe combined immunodeficiency (SCID) mice present spontaneous mutations on the kinase domain of the DNA-PK (*Prkdc*) gene that leads to a loss of kinase activity. These animals display immunodeficiency and DSB repair impairments, premature aging, and telomeric fusions [313,314,315]. Interestingly, while no significant neural phenotype was observed in *Prkdc*-null mice [316], DNA-PKcs (*scid/scid*) mice exhibit elevated neuronal apoptosis in the embryonic brain [317,318]. The simultaneous loss of polymerase β and *Prdkdc* showed increased growth arrest, neuronal apoptosis, and lethality, indicating an interaction between Polβ and DNA-PK during neurodevelopment [317]. Notably, these studies did not analyze the direct evidence of replication stress in NPCs. 

DNA-PK is also important in retinogenesis. In SCID-mice retinas, proliferating NPCs die after *Prkdc* loss. In addition, the pharmacological inhibition of DNA-PK in organotypic cultures induced caspase-dependent cell death and selectively affected the neurogenesis of early-born retinal types, indicating a possible role in the prevention of replication stress in retinal progenitor cells [319]. More recently, Enriquez-Rios and colleagues closely studied the cortical neurogenesis of *Prkdc*-null mice. While DNA-PK is required for NPC responses to irradiation-induced DNA damage, no role for DNA-PK in the protective mechanisms against DNA replication was observed [295].

### 5.2. The MRN Complex: Mre11, Rad50, and Nbs1

The MRN complex, composed of Mre11, Rad50, and Nbs1, is key for the detection and repair of DSBs and regulates multiple aspects of DDR. In addition to the initial detection of DSBs and stalled replication forks, this complex contributes to ATM activation and to signaling mediated by both ATM and ATR. The MRN complex also plays relevant roles in dysfunctional telomeres and in combating viral DNA. Therefore, crucial cellular responses such as cell cycle progression, the commitment to DNA repair pathways, and chromatin remodeling are regulated by the MRN complex. Distinct single-gene disorders illustrate the importance of the MRN complex components to the CNS development and function. Ataxia-telangiectasia-like disorder 1 (ATLD1) is caused by mutations in *MRE11A*, and Nijmegen breakage syndrome (NBS) is caused by NBS1 hypomorphism [320,321,322,323] (Table 1).

#### 5.2.1. Ataxia-Telangiectasia-Like Disorder 1 (ATLD1)

ATLD1 (OMIM #604391) is an autosomal recessive disorder that shares common clinical features with A-T patients (radiosensitivity, microcephaly, progressive ataxia, and cerebellar degeneration), except for telangiectases or immunodeficiency. It is caused by hypomorphic homozygous or compound heterozygous mutations in the meiotic recombination 11 homolog 1 gene (*MRE11A*) [324,325,326] (Table 1).

The two catalytic components of the MRN complex are MRE11, which encodes a nuclease with both exo- and endonuclease activities, and the RAD50 ATPase. Nucleolytic actions of MRE11 on dsDNA depend on the RAD50 activity; however, RAD50-catalyzed ATP hydrolysis is not essential for all MRE11 functions. NBS1, in turn, is a key modulator of MRE11 activities. In replicating forks, MRN-mediated resection is a key mediator of replication stress generation and RSR. While MRN nuclease activity helps to solve stalled replication forks, excessive resection can result in fork degradation [320,321].

A MRE11 allele that recapitulated ATLD patient hypomorphism led to a pronounced chromosomal instability and confirmed the Mre11 relevance for ATM activation and for early embryogenesis but did not report neurological phenotypes [327]. In cells, it was shown that components of the MRN complex are required to prevent MYC-induced replication stress in primary cultures of granule cells, the NPCs of the cerebellum [328].

Hypomorphic mutations in proliferating cell nuclear antigen (PCNA) were reported in one family with similar neurological features (ATLD2) [329]. An analysis of DNA replication and repair in patient fibroblasts indicated that DNA replication was not severely impaired, but these cells displayed high UV sensitivity. It was suggested that defective nucleotide excision repair (NER) caused the observed phenotypes. Currently, however, we do not know the effects of hypomorphic PCNA in cell proliferation or RSR in cells of the neural lineage.

#### 5.2.2. Nijmegen Breakage Syndrome (NBS)

Nijmegen breakage syndrome (NBS, OMIM #251260) is an autosomal recessive disorder caused by hypomorphic mutations of the *NBN* gene, leading to microcephaly, growth retardation, immunodeficiency, a predisposition to cancer, premature aging, and neurodegeneration. With the exception of cerebellar neurodegeneration and ataxia, clinical and cellular features of NBS overlap with A-T and ATLD [330,331] (Table 1).

The germline inactivation of the *Nbn/Nbs1* gene in mice led to an early embryonic lethality [332]. A murine model of the syndrome coding the mutated *Nbn* [333] confirmed the important role of DDR to embryogenesis and replicated a few disease phenotypes but did not fully elucidate the NBS1 roles in the nervous system development. The CNS-specific inactivation of NBS1 led to microcephaly, ataxia, and, different from human NBS patients, cerebellar degeneration. These phenotypes were caused by a p53-mediated arrest of progenitor proliferation and neuronal apoptosis. Later, other studies revealed that *Nbn* loss also compromised the visual system development, leading to a mild apoptosis of NPCs of the retina, demyelination of the optic nerves, and impaired retinal functions [334,335]. These studies also shed light in the functional interplay between NBS1 and ATM, revealing that, in the developing CNS, these proteins collaborate to prevent DSB accumulation and the apoptosis of progenitor cells in a tissue- and developmental stage-specific manner [335,336]. None of these studies interrogated the S-phase-specific or direct roles of NBS1 in the DNA replication of NPCs.

## 6. Non-Cell-Autonomous RSR in the CNS

In addition to cell-autonomous mechanisms activated by RSR, it is now clear that replication stress may also induce alterations in the cellular microenvironment. The disruption of replication forks and/or DNA repair processes may induce the accumulation of cytoplasmic DNA (either dsDNA, micronuclei, or “speckles”, ssDNA foci less aggregated than micronuclei), triggering the synthesis of cytokines and the activation of immune responses [19,20,80,337]. This non-cell-autonomous arm of the RSR is mainly mediated by the cyclic-GMP-AMP synthase-Stimulator of Interferon Genes (cGAS-STING) pathway.

**Figure 5:**
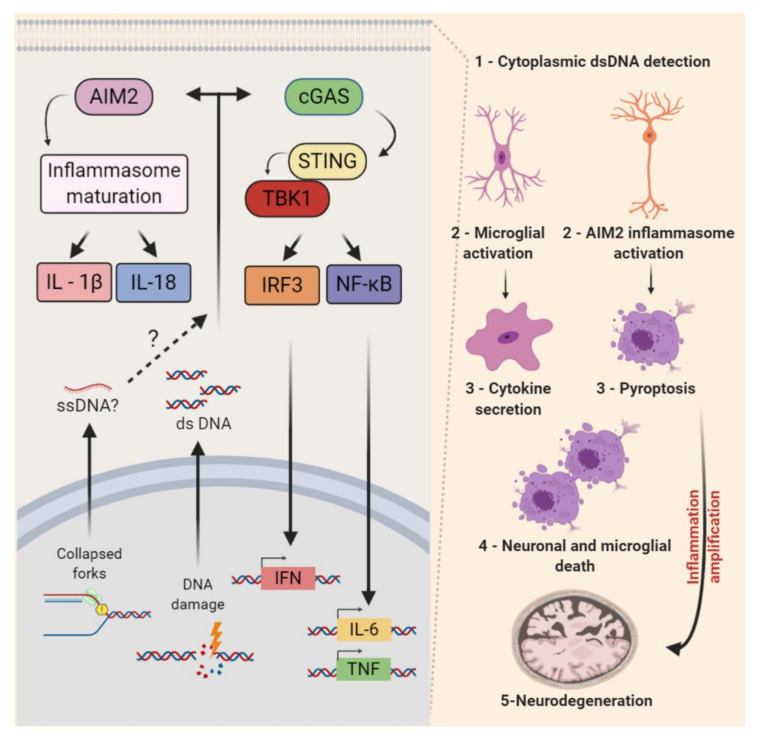
Nonautonomous replication stress response in the central nervous system. The detection of self-nucleotides (ssDNA, dsDNA, and RNA) in the cytoplasm can trigger the activation of two main proinflammatory signaling pathways. Cytoplasmic DNA may be recognized by cyclic-GMP-AMP synthase (cGAS), which leads to the activation of Stimulator of Interferon Genes (STING) that may activate TANK-binding kinase 1 (TBK1) and, consequently, its downstream targets IRF3 and NF-κB. These may stimulate the transcription of type I interferons (IFN), interleukin (IL)-6, and the tumor necrosis factor (TNF), among other proinflammatory factors. The AIM2 inflammasome also recognizes dsDNA (foreign or self) and activates caspase-1, leading to the maturation and/or externalization of proinflammatory interleukins (e.g., IL-1β and IL-18). The activation of this pathway can lead to pyroptosis. In the CNS, the activation of both pathways was reported in the microglia of *Atm* knockout mice. The cGAS-STING-mediated activation of microglia and an increased cytokine secretion were associated with chronic inflammation and neuronal cell death in the context of cerebellar neurodegeneration. The pronounced activation of AIM2 inflammasome in the context of DNA damage was also reported in the developing CNS. The inactivation of AIM2 decreased the developmental cell death and impacted adult mice behaviors. Whether the endogenous replication stress of NPCs contributes to the activation of AIM2 inflammasome remains to be determined.

Cyclic-GMP-AMP synthase (cGAS) is a well-characterized sensor of cytoplasmic DNA driving cytokine production. DNA binding to cGAS activates the synthesis of 2′ and 3′-cGAMP (cyclic guanosine monophosphate—adenosine monophosphate synthase) from ATP or GTP [338]. The second messenger cGAMP led to the oligomerization and activation of the adaptor protein STING (Stimulator of Interferon Genes). After its translocation to the Golgi apparatus, STING activates TBK1 (TANK-binding kinase 1) and its downstream targets: Interferon regulatory factor 3 (IRF3) and the canonical or noncanonical Nuclear factor kappa B (NF-κB) pathways. Then, transcriptional regulators IRF3 and NF-κB may induce the expression of type I interferon (IFN) and other proinflammatory cytokines [339,340,341]. In various biological contexts, it has been reported that the exposure to replication stress-inducing agents and defective RSR activates inflammatory responses [342,343]. A few studies indicate that cGAS-STING may also play relevant roles in microglia-induced neurodegeneration. In addition, a recent study indicated that Absent in melanoma 2 (AIM2), also a sensor of cytoplasmic DNA, may regulate the CNS development [344] (Figure 5).

The progressive neurodegeneration that affects mostly the Purkinje and granule cells of the cerebellum is a major issue in A-T disease. Many evidences point that the neurodegeneration associated with ATM deficiency can be influenced by pathologic inflammation [322] and interestingly, the cGAS-STING pathway may clarify this link. First, Härtlova and colleagues reported that spontaneous DNA lesions accumulate and a STING-dependent induction of IFN in fibroblasts and immune cells of *Atm* null mice [345]. In the nervous system, it was observed that LPS-induced inflammation in vivoS aggravated A-T neurological phenotypes and induced the degeneration of ATM-deficient Purkinje cells [346]. Moreover, ibuprofen, a non-steroidal anti-inflammatory drug, inhibited microglial activation and Purkinje cell damage in *Atm* null mice [347]. A direct link of defective DDR, cGAS-STING pathway, inflammatory cytokines, and neurodegeneration was recently established. In *Atm* ^−/−^ mice: *Atm*-deficient microglia accumulate cytoplasmic DNA and trigger a STING-mediated proinflammatory response that secretes neurotoxic interleukins [81] (Figure 5). Consistent findings were also reported in a rat model of A-T [348].

Interestingly, the pharmacological inhibition of ATM activated the AIM2 inflammasome in cultured microglial cells [81]. Recently, evidence that this cytoplasmic DNA sensor is a key regulator of CNS development has also emerged. The genetic inactivation of AIM2 decreased neuronal cell deaths during development, increased the neuronal numbers in the adult brain, and impacted mice behaviors. Even though it was not determined whether endogenous replication stress was the source of damaged DNA, these findings revealed that the AIM2 inflammasome mediates the removal of cells containing DNA damage from the developing brain [344]. Altogether, these studies opened new avenues of investigation into the functional relationship between non-autonomous RSR and neuroinflammation in the CNS development and degeneration.

## 7. Potential Contributions of Replicative Stress to Genomic Variations in the Nervous System

Seminal studies about XRCC4 and ligase 4 established a critical role of a nonhomologous end joining (NHEJ) type of DSB repair for proper neurogenesis and for the prevention of CNS tumorigenesis. The germline inactivation of XRCC4 or DNA ligase 4 led to similar phenotypes: late-embryonic lethality due to neural development defects caused by unrepaired DSBs and the p53-mediated apoptosis of NPCs [349,350]. As observed in the immune system, the conditional inactivation of XRCC4 and p53 led to brain tumors (medulloblastoma) with recurrent translocations on different chromosomes and frequent chromosomal amplifications [351].

In subsequent studies, it was asked whether the absence of DSB repair proteins in NPCs would allow the identification of endogenous sequences prone to recurrent DSBs. Applying a high throughput technology of detection of translocating sequences genome-wide (HTGTS) to cultured XRCC4 and p53-double-deficient NPCs, Wei and colleagues characterized a massive number of translocated DSBs in these NPCs [17,352]. The genome-wide search for clusters of DSBs based on the translocation of genomic sequences to ectopically generated bait DSBs on several different chromosomes allowed the identification of 27 recurrent DSB clusters in the genome of NPCs. Two aspects of these findings strongly indicate that replicative stress contributes to the generation of these breaks in NPCs. Many of the translocated DSBs were observed in wild-type NPCs, and the induction of mild replication stress (treatment with aphidicolin) enhanced the phenomenon. Furthermore, most of the DSB clusters were localized to long- and late-replicating genes [17,353]. These particularities (replication in the late-S-phase and a sensibility to replication stress-inducing agents) indicated TRCs as a possible causative agent of the structural variances [16,354,355]. The evidence that hotspots for common fragile sites (CFS) and copy number variations (CNVs) were also detected in long- and, often, late-replicating genes reinforced the link between TRCs and structural genomic variations. Of relevance for neural functions, one-third of these recurrent DSB clusters traced back to regions where CNVs were detected in human NPCs [356]. In addition, all sites of recurrent DSBs lied within genes that encoded for proteins involved in synaptogenesis and other neural processes. Some of these genes have already been associated with neurological disorders (e.g., schizophrenia and autism) and brain cancers [357].

The presence of cells containing genomic variations distinct from the germline is defined as somatic mosaicism [358]. Brain mosaicism has been elegantly demonstrated, and implications for neurodevelopmental, neurodegenerative, and even psychiatric diseases have been suggested [358,359,360,361]. Although extensive investigation is still required, it is possible that replication stress contributes to the generation of genomic structural variations and to mosaicism during neurogenesis and in the adult brain. For instance, single-nucleotide variations (SNVs) constitute another type of frequent genomic alteration in the mature brain. About 200–400 somatic SNVs have been detected per NPC, with the number reaching around 1000 to 2500 in adult individuals [362,363]. SNVs increase gradually with age, and the increase appears to be proportional to the rate of neurogenesis in specific brain regions. The dentate gyrus, a site of adult neurogenesis (Figure 4), may reach 40 events/year [364]. The higher index of variations in these sites of persistent cell proliferation might suggest a correlation between replication stress and the generation of SNVs. Some studies presented associations between these SNVs variations and neurological diseases [365,366,367]. Finally, a lingering question in the field is whether the different examples of genomic mosaicism described in the CNS contribute to the pathogenesis of neurodevelopmental disorders and neurodegenerative diseases.

## 8. Conclusions

The rapid expansion of progenitor cell pools during development, the extremely high metabolic activity, and the long-lived nature of neurons and glia may explain the high vulnerability of the nervous system to defective DDR. The findings discussed in this review reinforce the relevance of DDR and highlight how cell-autonomous and non-cell-autonomous replication stress responses (RSRs) contribute to the nervous system development, physiology, and neurological diseases. Recent findings expanded our knowledge about the importance of a proper prevention of replication stress during neurodevelopment. Moreover, they highlighted crucial novel mechanisms of protection against DNA replication stress in mature CNS functions (adult neurogenesis) and in neurodegenerative diseases. The elucidation of the specificities of RSR in distinct biological contexts (e.g., specific tissues, developmental stages, and diseases) are still necessary. Further studies about the role of RSR in neuronal tissues may contribute to the prevention and to the development of therapies for neurological disorders.

## Figures and Tables

**Table 1:** Human neurologic syndromes and mutations in genes related to replication stress (RS).

Syndrome	OMIM	Mutated Genes	Mechanisms Described	Neuropathology
Seckel Syndrome (SS)	210600	*ATR, ATRIP*	Replication stress response	Microcephaly, cortical and retinal malformations
Ataxia telangiectasia (A-T)	208900	*ATM*	DSB signaling, Replication stress	Neurodegeneration
Ataxia-telangiectasia-like disorder 1 (ATLD1)	604391	*MRE11*	DSB signaling, Fork resection	Ataxia, neurodegeneration, dysarthria, oculomotor apraxia
Nijmegen Breakage Syndrome (NBS)	251260	*NBN*	DSB signaling	Microcephaly
Fanconi Anemia (FA)	605724 227646	*BRCA2, FANCD2*	DNA crosslink repair Fork protection	Medulloblastoma, microcephaly and hydrocephalus
Aicardi-Goutières Syndrome (AGS)	225750 610181 610329 610333 612952 615010	*TREX1* *RNASEH2B* *RNASEH2C* *RNASEH2A* *SAMHD1* *ADAR1*	Removal of DNA:RNA hybrids, Ribonucleotide excision, dNTP hydrolysis, RNA editing	Microcephaly, cerebral atrophy, demyelination
Werner Syndrome (WRN)	277700	*WRN*	Complex fork structure resolution	Brain atrophy, memory deficits
Immunodeficiency 26 (IM26)	615966	*PRKDC*	DSB signaling	Brain atrophy, hypomyelination, visual impairment, hearing loss
Spinocerebellar ataxia, autosomal recessive 26 (SCAR26)	617633	*XRCC1*	Scaffold protein	Ocular motor apraxia, progressive cerebellar atrophy
Spinocerebellar ataxia, autosomal recessive, with axonal neuropathy 2	606002	*SETX* (AR) *	DNA/RNA helicase	Cerebellar ataxia, axonal neuropathy, oculomotor apraxia
Amyotrophic lateral sclerosis 4	602433	*SETX* (AD) *	DNA/RNA helicase	Spinal cord atrophy, hyperreflexia, axonal neuropathy

* AD—autosomal dominant and AR—autosomal recessive.
